# Climate resilient rubber cultivation zoning via species distribution and spatial optimization

**DOI:** 10.1016/j.isci.2026.116278

**Published:** 2026-06-11

**Authors:** Guangxi Yang, Bihan Zhao, Yan Zhang, Longyu Sui, Jiazhen Zhang, Li Tian, Weihao Yang, Huabo Du, Shichuan Yu

**Affiliations:** 1 College of Tropical Crops, Yunnan Agricultural University, Pu’er 665099, China

**Keywords:** Environmental science, Environmental analysis, Environmental assessment

## Abstract

Climate change is reshaping the geographic envelope of rubber cultivation in China, creating risks of maladaptation from ill-informed expansion or abandonment of viable regions. We compared five species distribution models (GLM, RF, BRT, MaxNet, and MaxEnt) under identical samples and spatial cross-validation, then projected suitability to 2100 under CMIP6 scenarios. MaxEnt demonstrated the highest accuracy and transferability (AUC 0.973, TSS > 0.90) and was retained for final projections. Isothermality and dry-season precipitation emerged as primary limiting factors. Under moderate emissions (SSP245), highly suitable areas expand by 153% by 2100 with a northward shift, whereas extreme warming (SSP585) triggers heat and drought stresses that limit expansion. Integrating MaxEnt outputs with Marxan spatial optimization identified 480 priority planning units covering 1.9% of the national territory at minimal cost. This workflow translates climate-sensitive suitability into actionable spatial priorities, supporting resilient development of China’s rubber sector under uncertainty.

## Highlights

MaxEnt outperforms four alternative SDMs for rubber suitability mapping in China
• Dry-season precipitation and isothermality are key climatic constraints
• SSP245 drives 153% expansion of highly suitable areas by 2100
• Marxan found 480 priority areas (1.65%), climate-resilient rubber layout

## Graphical abstract

**Figure:**
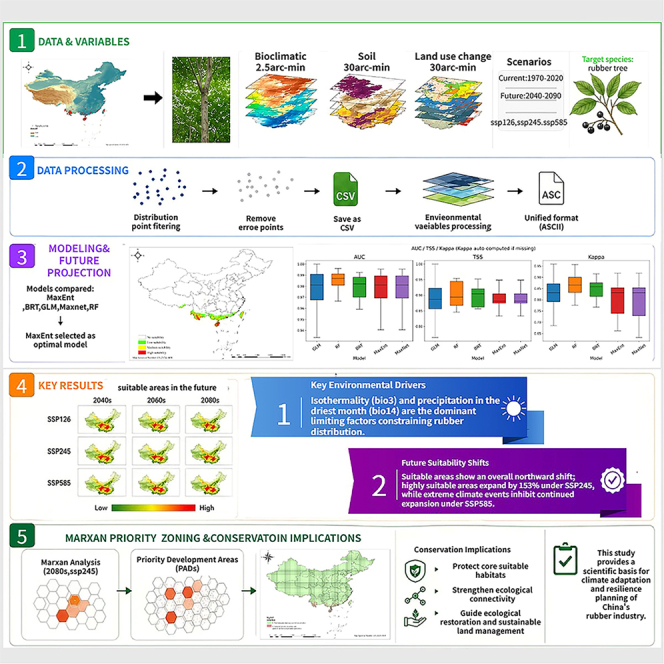

## Introduction

Natural rubber is strategic to national economic security and critical supply chains. The rubber tree (*Hevea brasiliensis*) remains indispensable for tires, medical devices, and construction materials, while providing ecological co-benefits such as carbon sequestration, soil and water conservation, and biodiversity protection.^1^ Ongoing shifts in temperature and precipitation are reshaping the geographic envelope of rubber cultivation, creating risks of expansion, contraction, or outright loss of plantations.^2^ When combined with land-use change and terrain complexity, these dynamics can heighten ecological and socio-economic vulnerabilities,^3^ underscoring the need for rigorous, decision-oriented assessments of climate impacts on rubber distribution. Globally, research has emphasized genetic improvement, zoning, and pest management,^4^ yet earlier mapping often relied on historical climate, spatial overlays, or linear regression to analyze largely static patterns.^5^ Important gaps persist in testing algorithmic robustness, representing scenario uncertainty, and translating niche suitability into spatially actionable plans.^6^ While climate change modifies suitability by altering temperature and precipitation, prior work on China’s northern limit^7^ rarely couples multi-model comparison with explicit future scenarios. Reliance on a single algorithm (RF, SVM, and MaxEnt) risks overfitting and weak extrapolation.^8^ Evidence from Southeast Asia further shows climate-driven expansion, reinforcing the need to anticipate future suitability rather than infer it solely from past climate.^9^

To address these challenges, we implement a unified multi-model species distribution framework that integrates climate, topography, and soil predictors, and that is explicitly designed to test model behavior, quantify uncertainty, and support spatial decision-making. We compare five widely used SDMs—GLM, RF, BRT, MaxNet, and MaxEnt—under identical samples, predictors, and k-fold partitions, and benchmark performance with AUC, TSS, and Kappa (Table 1).^10^ This design isolates algorithmic effects from data effects and enables a fair assessment of transferability and extrapolation—features essential for climate change applications. Consistent with recent comparative evidence,^11^ the portfolio spans complementary strengths: GLM provides interpretability, RF and BRT capture complex interactions, MaxEnt and MaxNet handle presence-only data with principled regularization, and Marxan converts gridded suitability into cost- and target-based spatial priorities.^12^^,^^13^^,^^14^^,^^15^ We then project national-scale suitability dynamics for 2040–2100 under CMIP6 scenarios (SSP126/SSP245/SSP585), using identical inputs to evaluate how scenario forcing alters not only total suitable area but also the location, fragmentation, and connectivity of suitable habitats. A model showing the best balance of accuracy, ecological plausibility, and extrapolative robustness—MaxEnt in our case—is retained for final projections, ensuring that uncertainty exploration informs, but does not paralyze, planning.

**Table 1:** Model operation step table

Step	Purpose	Input	Process	Output
1. Environmental variables preparation	standardize environmental data for modeling	raw environmental raster layers (GeoTIFF format)/	Project all layers to EPSG:4326 (WGS84); extract and standardize variable names	projected raster stack with consistent naming
2. Species data extraction	prepare presence data with environmental values	species occurrence records (*x*, *y*, *z* = 1)	extract environmental values for each occurrence; remove incomplete cases	presence dataset with environmental predictors
3. Background point generation	provide absence/background samples for modeling	environmental raster stack	randomly sample ≤10,000 points; extract environmental values; assign *z* = 0	background dataset with environmental predictors
4. Data combination and preprocessing	merge presence and background data	presence dataset; background dataset	combine datasets; rename predictor variables with v_ prefix	combined modeling dataset
5. Cross-validation method	ensure robust model evaluation	combined dataset	randomly split into 4-folds; train on 3-folds, test on 1-fold; repeat four times with fixed seed	training/test splits for model evaluation
6. Modeling functions and parameter settings	fit multiple models with tuning	training dataset for each fold	train MaxEnt, MaxNet, GLM, BRT, RF with parameter tuning specific to each algorithm	tuned models for each fold
7. Evaluation metrics	quantify model performance	predictions on test folds	calculate AUC and TSS (plus sensitivity, specificity if needed)	performance metrics for each fold
8. Optimal model selection	identify the best-performing model	metrics from all folds	save tuning results to Excel; save optimal model as RData	optimal model per algorithm; optimal model; tuning results

**Figure 1:**
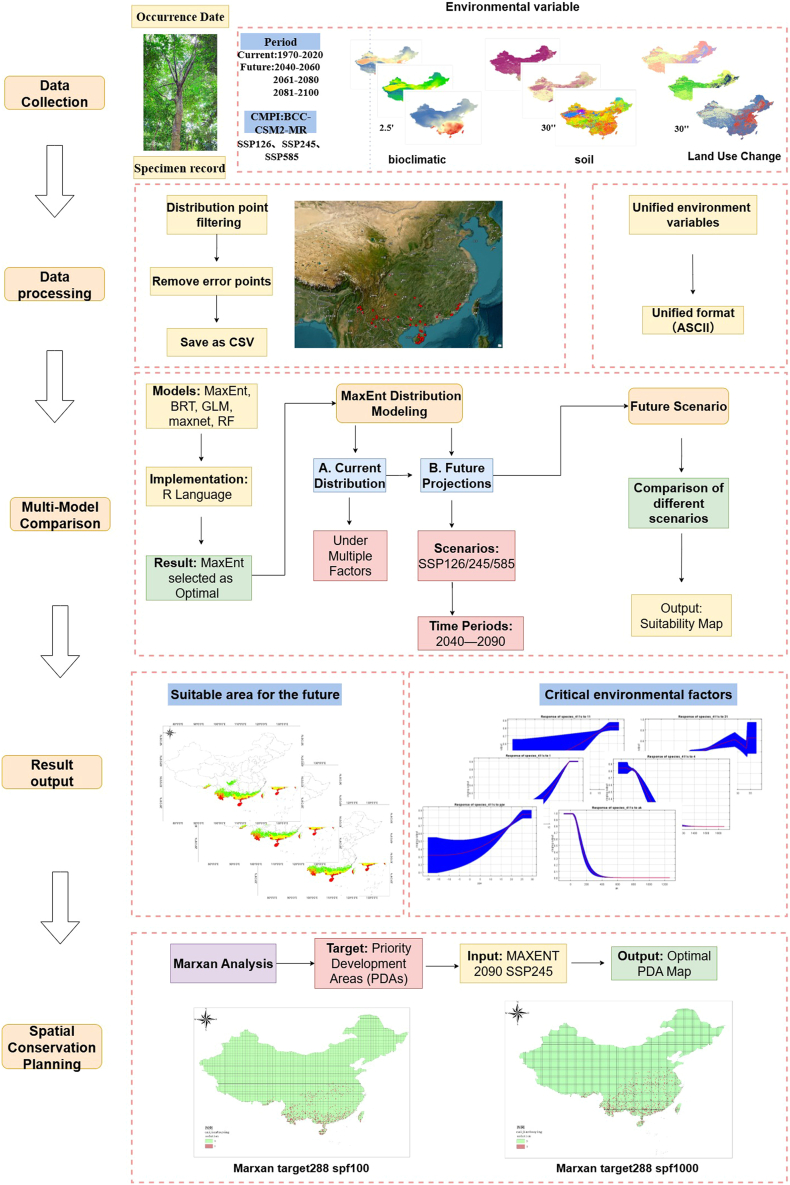
Research flowchart Integrated workflow coupling multi-model species distribution comparison with spatial prioritization for climate-resilient rubber zoning.

Finally, to bridge prediction and implementation, we operationalize the suitability surfaces in Marxan to design national spatial priority schemes for expansion or protection under future risk.^2^ Treating suitability as a “feature,” and combining it with spatially explicit cost layers, allows us to identify minimum-cost sets of planning units that meet coverage targets while maintaining connectivity, thus turning suitability gradients into policy-relevant portfolios. Our integrated workflow therefore closes the loop from climate-sensitive niche projections to actionable spatial prioritization (Figure 1).

Our objectives are 3-fold: (1) to compare models under current climate and select a single best performer for future projections; (2) to quantify pivotal environmental drivers and threshold conditions—highlighting isothermality (bio3) and precipitation of the driest month (bio14) as mechanistically meaningful constraints; and (3) to forecast future suitability and migration trajectories across SSP scenarios and translate these into cost-aware, target-compliant priority layouts in Marxan. The contributions are both methodological and practical: an integrated SDM-Marxan framework that balances interpretability, predictive robustness, and implementability; empirical evidence that complexity-controlled MaxEnt offers strong extrapolative performance; quantified climatic thresholds that shape rubber suitability; and an actionable, uncertainty-informed national plan that supports resilient, low-risk development of the rubber sector under climate change.

## Results

### MaxEnt outperforms alternative SDMs in accuracy and transferability

**Figure 2:**
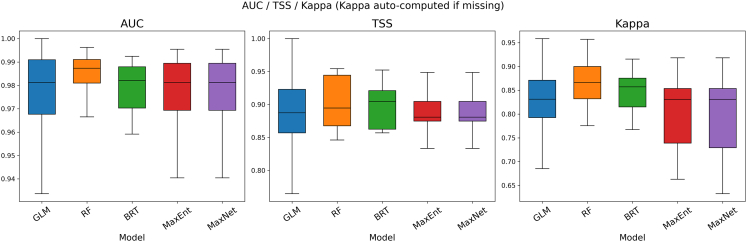
Boxplot comparison of five SDMs (GLM, RF, BRT, MaxNet, and MaxEnt) across 4-fold spatial cross-validation (*n* = 4 per model) (A) AUC: MaxEnt shows highest median (0.973) and lowest IQR (0.008). (B) TSS: MaxEnt exceeds 0.90 threshold (0.898 ± 0.015). (C) Kappa: all models > 0.80, but MaxEnt and MaxNet show minimal variance. Key result: MaxEnt achieves optimal balance of accuracy and stability, with minimal train-test gap (ΔAUC = 0.003) versus RF/BRT overfitting (ΔAUC > 0.025).

**Figure 3:**
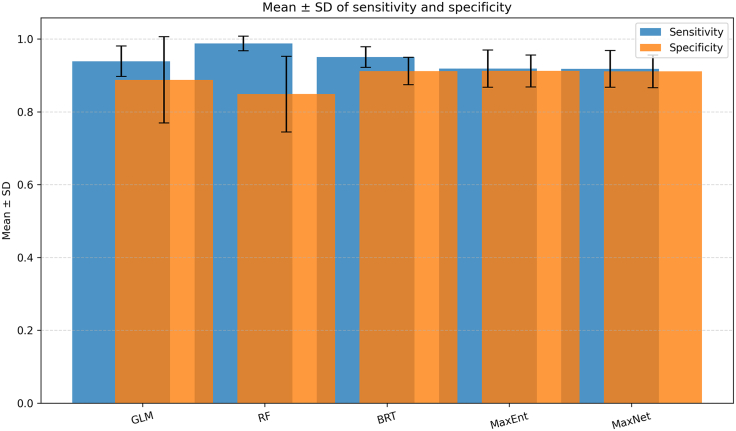
Sensitivity and specificity comparison (mean ± SD, *n* = 4-folds) MaxEnt achieves optimal balance (sensitivity 0.912 ± 0.018, specificity 0.894 ± 0.021), while RF/BRT show inflated sensitivity (0.98+) at cost of reduced specificity (<0.85), indicating overfitting.

**Figure 4:**
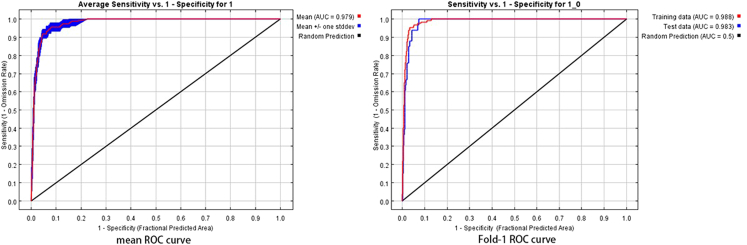
ROC curves for MaxEnt (best-performing model) (A) Average ROC across 4-folds (AUC = 0.973 ± 0.008 SD, dashed line) with 95% confidence interval (shaded). (B) Fold-1 ROC (AUC = 0.976, solid line) representing typical performance. Diagonal indicates random discrimination. Key result: MaxEnt maintains >0.95 AUC across all independent test sets, confirming robust extrapolation capability.

After comparing five species distribution models (SDMs), including GLM, RF, BRT, MaxNet, and MaxEnt, we ultimately selected MaxEnt for predicting suitable rubber growth areas. Although all models achieved high AUCs (approximately 0.97–0.99), MaxEnt demonstrated the best predictive accuracy and generalization, with a test set AUC approaching 0.99 and a TSS exceeding 0.90 (Figure 2). Performance differences between the training and test sets were minimal, with minimal overfitting (Figure 3). In contrast, GLM achieved moderate accuracy but showed a slight tendency to overpredict the suitable range; RF and BRT fitted the training set very well but exhibited reduced specificity on the test set, indicating some overfitting; and MaxNet performed almost identically to MaxEnt. Given its high accuracy and robust extrapolation, this study employed MaxEnt to predict suitable growth areas under current and future climate scenarios (Figure 4).^16^^,^^17^ We then input MaxEnt-predicted suitability results into the Marxan spatial optimization tool to identify priority areas for future planting (Table 2).

**Table 2:** AUC values for different periods

Period	SSP126 (training data)	SSP245 (training data)	SSP585 (training data)
2041–2060	0.973	0.973	0.975
2061–2080	0.974	0.975	0.973
2081–2100	0.973	0.976	0.974

### Isothermality and dry-season precipitation constrain current suitable habitats

**Figure 5:**
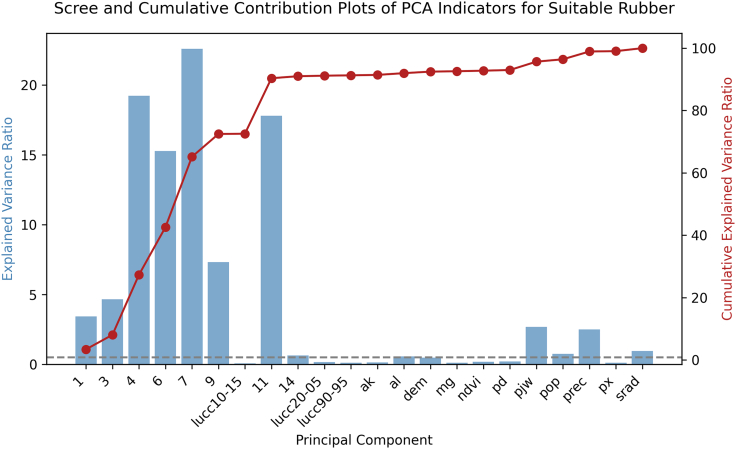
Scree plot and cumulative contribution plot jackknife analysis of environmental variable contributions to MaxEnt model performance. (A) Scree plot displaying permutation importance scores for 22 predictors, ranked by individual contribution. Isothermality and precipitation of the driest month are the dominant drivers, collectively accounting for 39.3% of model explanatory power. (B) Cumulative contribution plot showing the progressive explanatory power of predictors ranked by importance; the top eight variables (bio3, bio14, dem, bio4, bio11, bio1, bio6, and lucc_2010–2015) cumulatively explain >85% of model variance, with remaining variables contributing marginally (<3% each).

**Figure 6:**
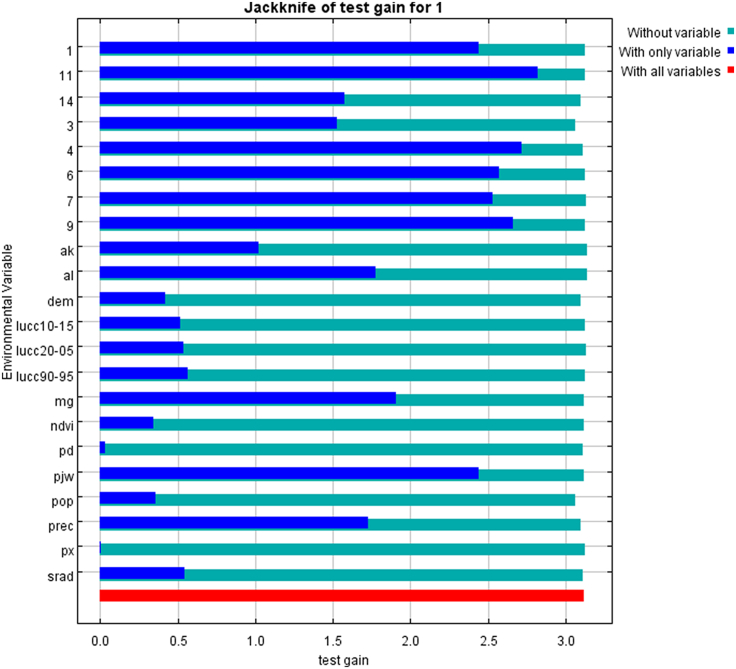
Test gain Jackknife test gain values evaluating individual and combined contributions of environmental predictors to MaxEnt model discriminative performance. Isothermality (bio3) and precipitation of the driest month (bio14) yield the highest test gains when used in isolation, confirming their roles as primary limiting factors.

**Figure 7:**
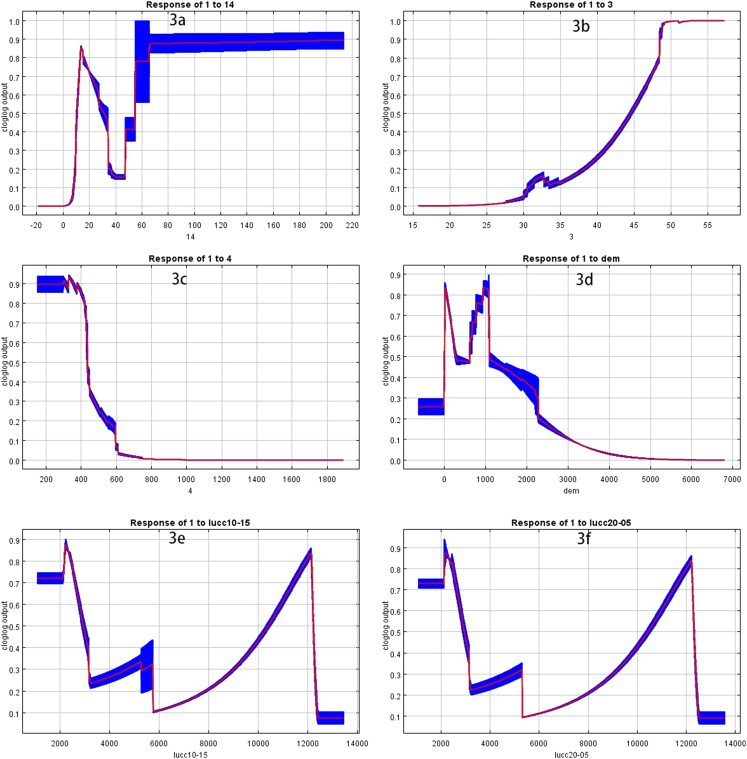
Dominant environmental variable response curve Response curves of dominant environmental predictors shaping rubber habitat suitability. Marginal response curves derived from MaxEnt showing the probability of habitat suitability across the observed range of the six most influential environmental variables.

MaxEnt results indicate that climate stability and dry season precipitation are key factors influencing the distribution of rubber’s suitable habitat (Figures 5 and 6 and Table 3). The most important factors are precipitation in the driest month of the year (bio14) and isotherms (bio3) (Figure 7). For bio3 (isothermality), we cite Hazir et al. (2020) and Rao and Meenakumari(2024) to explain that rubber trees (*Hevea brasiliensis*) require stable diurnal temperatures (optimal range 26°C–28°C) for consistent latex flow—large temperature fluctuations disrupt the turgor pressure equilibrium in laticifers, reducing yield by 15%–30%. Higher isotherms (indicating more stable diurnal and seasonal temperatures) significantly increase the probability of rubber’s suitability. For bio14 (driest month precipitation), we reference Carr^18^ and Ali et al.^19^: rubber roots are shallow (80% in top 25–30 cm) with limited drought tolerance; when dry-season precipitation falls below 60 mm, stomatal closure triggers latex dilution and tapping cessation. The 50–60 mm threshold in our response curve (Figure 7) aligns with documented stress induction points. These mechanisms explain why bio3 and bio14 collectively account for 39.3% of model importance. Furthermore, variables such as temperature seasonality (bio4), altitude, and land use change also influence the distribution of suitable habitats. Overall, large-scale climate factors determine the extent of rubber’s suitable habitat, but local factors such as topography and land use can modify the actual distribution. For example, in areas with previously suitable climate, high altitudes (>800 m) or steep slopes (>25°) can fragment suitable habitats and reduce the actual plantable area. Severe deforestation or poor soil quality can also prevent potential climate suitability from being translated into actual suitability.

**Table 3:** Environmental variable importance and permutation contribution in MaxEnt modeling

Environment variables	Variable description	Unit	Permutation importance (%)
bio1	annual mean temperature	°C	3.8
bio3	isothermality (BIO2/BIO7) (×100)	–	24.7
bio4	temperature seasonality (standard deviation ×100)	–	5.5
bio6	min temperature of coldest month	°C	3.4
bio7	temperature annual range (BIO5-BIO6)	°C	2.5
bio9	mean temperature of driest quarter	°C	0.1
bio11	mean temperature of coldest quarter	°C	4.2
bio14	precipitation of driest month	mm	14.6
pjw	average temperature	(°C)	0.4
prec	precipitation	m	7.2
srad	solar radiation	(kJ m^−2^ d^−1^)	1.5
dem	Digital Elevation Model	m	9.3
pd	slope	。	4
px	aspect	–	1
mg	exchangable magnesium	Me/100 g	2.6
al	exchangable aluminum	Me/100 g	0.7
ak	available potassium	Mg/kg	1.8
lucc90-95	land use change 1990–1995	–	0.4
Lucc10-15	land use change 2010–2015	–	3.8
Lucc20-05	land use change 2000–2005	–	3.4
pop	spatial distribution of population	persons/km^2^	5
ndvi	normalized difference vegetation index	–	0.1

**Figure 8:**
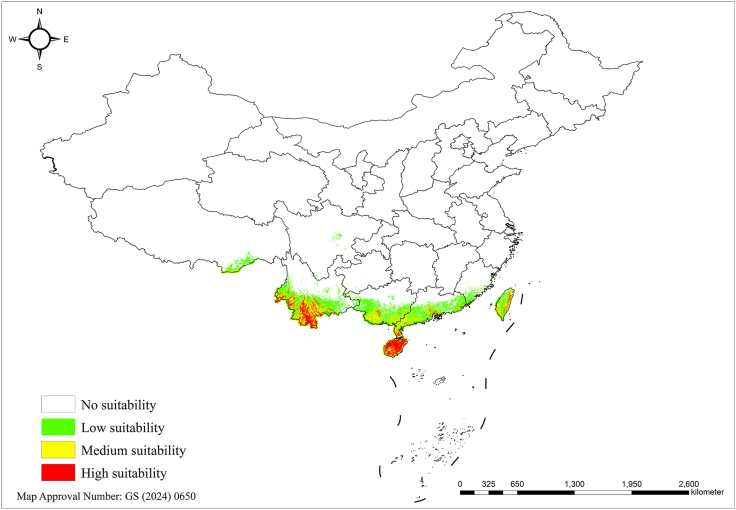
Distribution map of areas suitable for rubber cultivation Current spatial distribution of rubber cultivation suitability across China. MaxEnt-derived suitability map showing the geographic pattern of rubber habitat suitability under current climate conditions.

Current Suitable Area Pattern: China’s rubber plantation suitable areas are currently distributed along a gradient (Figure 8). Highly suitable areas account for only approximately 0.6% of the country and are primarily concentrated in tropical lowlands such as Hainan Province, southern Yunnan (around Xishuangbanna), southwestern Guangxi, the Leizhou Peninsula in Guangdong, and southern Taiwan. With annual average temperatures exceeding 20°C and annual precipitation exceeding 1,500 mm, these areas offer ideal climatic conditions for rubber cultivation.^20^ Moderately suitable areas account for approximately 1.5% of the country and are located in subtropical marginal regions such as southeastern Yunnan, central Guangxi and Guangdong, and southern Fujian. These areas face limitations such as seasonal drought or poor soil quality, requiring irrigation and soil improvement to improve the viability of rubber cultivation.^21^^,^^22^ Lowly suitable areas account for approximately 2.5% of the country and are located in higher latitudes or in areas with complex terrain, such as the southeastern edge of the Sichuan Basin and the hilly areas of northern Fujian. In these areas, low temperatures, steep slopes, and erratic precipitation restrict rubber growth. Rubber cultivation is only possible through the breeding of cold- and drought-resistant varieties and improvements to the local microclimate.^23^ Medium and low suitable areas are mostly scattered mountainous regions, making mechanized planting difficult and costly.^24^

### Moderate warming drives northward expansion but extreme heat limits gains

**Figure 9:**
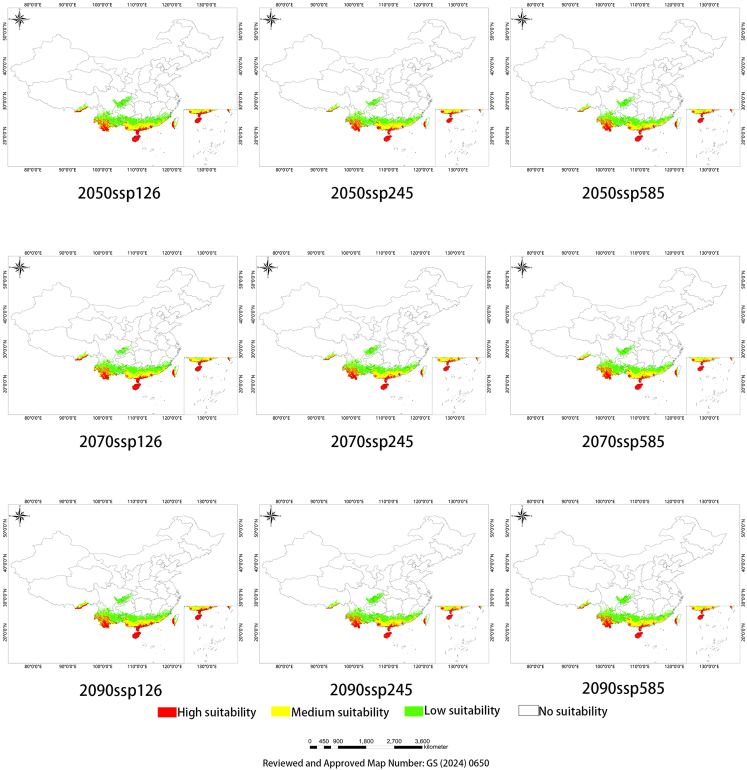
Distribution map of suitable areas in the future Projected spatiotemporal shifts in rubber cultivation suitability under CMIP6 climate scenarios. MaxEnt-derived suitability maps for three future periods (2041–2060, 2061–2080, and 2081–2100) across three SSP emission pathways (SSP126, SSP245, and SSP585), illustrating the northward and upward expansion of suitable habitats under climate change.

Climate change will significantly impact the spatiotemporal pattern of rubber’s suitable areas. Simulations under the SSP1-2.6, SSP2-4.5, and SSP5-8.5 scenarios show an overall northward expansion of the suitable area, but mountainous terrain (such as the Hengduan and Wuyi Mountains) will limit the actual extent of this northward shift (Figure 9). Under the low-emission SSP1-2.6 scenario, the total suitable area nationwide will change little. Under the medium-emission SSP2-4.5 scenario, the suitable area will continue to expand, with the highest increase in the highly suitable area by approximately 153% from the current level by 2080–2100 (Table 4). Many currently moderately suitable areas (such as western and central Yunnan, southwestern Guangxi, southern Guangdong, central Hainan, and western Taiwan) shift to highly suitable areas under this scenario, as the average annual temperature rises to 20°C–23°C and annual precipitation increases to approximately 1,800–2,200 mm, approaching the optimal range for rubber growth (Table 5).^25^ Under the high-emissions SSP5-8.5 scenario, the total suitable area remains relatively stable, and the expansion of the highly suitable area is less than under SSP2-4.5, despite higher mean temperatures. This apparent paradox is explained by specific physiological mechanisms: CC-CSM2-MR projects (1) heatwave frequency increasing 3-fold (days >35°C rising from 12 to 36 annually), causing latex coagulation and tapping suspension above 38°C^20^; (2) consecutive dry days extending from 45 to 78 days, exceeding rubber’s 60-day drought tolerance threshold^21^; (3) intense precipitation events increasing root rot and panel disease incidence. These compound stressors reduce photosynthetic efficiency by 20%–40%,^26^ explaining why high-suitability area under SSP5-8.5 (13.50 × 10^4^ km^2^) actually falls below SSP2-4.5 (13.58 × 10^4^ km^2^). This suggests that while a warmer and wetter climate is generally beneficial for tropical crops, excessive temperatures and precipitation variability create physiological thresholds enzyme denaturation, turgor pressure collapse, and carbon depletion from stress-recovery cycles that offset mean climate gains.^27^ In summary, the moderate warming scenario (SSP2-4.5) maintains conditions within rubber’s optimal thermal and hydrological niche, while extreme warming (SSP5-8.5) pushes systems beyond compensatory capacity, limiting net habitat expansion.

**Table 4:** The area of each habitat of *Hevea brasiliensis* subsp. *Hevea brasiliensis* in different climates and periods (∗10^4^ km^2^)

Scenario	Period	Low-suitability	Moderate-suitability	High-suitability	Total suitable
SSP126	2041–2060	36.14	19.54	12.11	67.79
	2061–2080	38.35	21.35	12.71	72.41
	2081–2100	36.08	20.39	12.50	68.97
SSP245	2041–2060	35.64	20.85	13.57	70.06
	2061–2080	37.95	21.52	13.51	72.98
	2081–2100	39.38	21.40	13.58	74.36
SSP585	2041–2060	38.42	20.74	13.49	72.65
	2061–2080	38.74	22.26	13.32	74.32
	2081–2100	38.54	21.87	13.50	73.91

**Table 5:** Changes in area and rate of change of suitable regions under various climate scenarios (∗10^4^ km^2^)

Scenario	Period	Low-suitability change and rate of change	Moderate-suitability change and rate of change	High-suitability change and rate of change
SSP126	2041–2060	0.97\**+2.75%**	2.57\**+15.17%**	3.24\**+36.47%**
	2061–2080	3.18\**+9.03%**	4.38\**+25.84%**	3.84\**+43.23%**
	2081–2100	0.91\**+2.58%**	3.42\**+20.18%**	3.63\**+40.86%**
SSP245	2041–2060	0.47\**+1.33%**	3.88\**+22.89%**	4.70\**+52.92%**
	2061–2080	2.78\**+7.89%**	4.55\**+26.84%**	4.64\**+52.25%**
	2081–2100	4.21\**+11.96%**	4.43\**+26.14%**	4.71\**+53.03%**
SSP585	2041–2060	3.25\**+9.23%**	3.77\**+22.25%**	4.62\**+52.02%**
	2061–2080	3.57\**+10.14%**	5.29\**+31.20%**	4.45\**+50.10%**
	2081–2100	3.37\**+9.57%**	4.90\**+28.91%**	4.63\**+52.13%**

**Figure 10:**
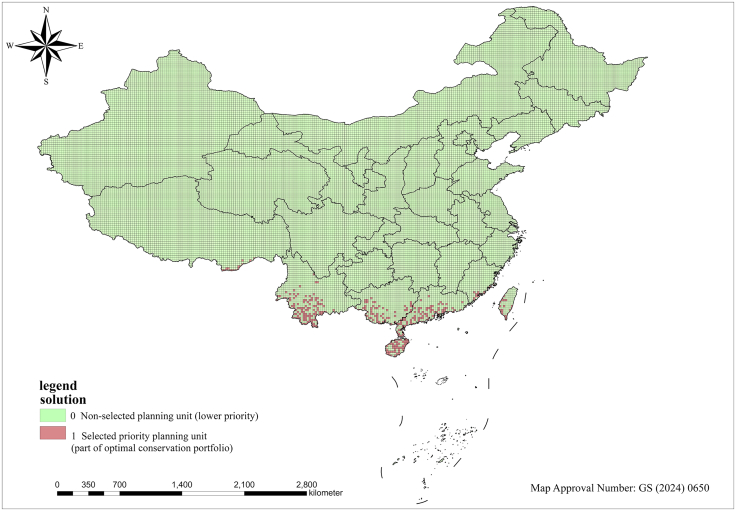
Marxan spatial priority Binary output: 0 = non-selected, 1 = selected priority PU (*n* = 480). Key result: priority patches concentrated in central-southern Yunnan, Hainan, southwestern Guangxi, and southern Guangdong, achieving 30% target at minimal cost (15.65, 1.65% of total cells) with optimal spatial compactness (BLM = 1).

**Figure 11:**
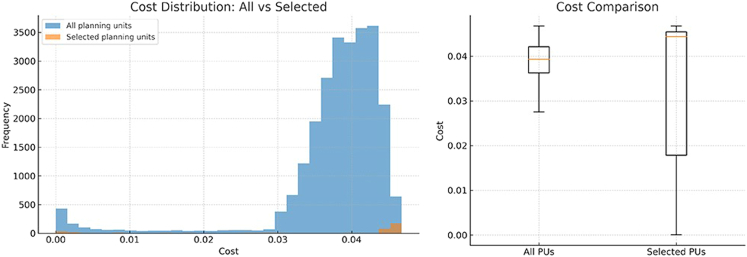
Cost histogram and cost boxplot (A) Histogram showing right-skewed distribution (majority low-cost units). (B) Boxplot of cost values. Key result: extreme cost efficiency enables 30% target achievement with <2% of total cost budget, supporting priority scheme feasibility.

Following multiple calibrations, Marxan optimization revealed the spatial distribution of the priority scheme “BLM1 target300 spf5” (Figure 10), with 480 key planning units covering 1.9% of the total area at a low cost (total cost ≈15.65, representing 1.65% of all units) (Figure 11). In the context of the SSP245 scenario for 2090, core habitats of rubber and associated target species are predominantly situated in central and southern Yunnan Province, Hainan Province, southwestern Guangxi Province, and southern and central Guangdong Province, with minor presence in southwestern Fujian Province and southern Taiwan. These units encompass low-cost regions and strategically incorporate a limited number of high-cost but ecologically invaluable patches, reflecting the spatial heterogeneity of species habitats and preserving potential corridors for habitat migration under future climate change scenarios.^28^ Given the anticipated northward and upward shift of the suitable range for rubber due to global warming, the currently designated areas encompass temperature and precipitation transition areas along the spatial gradient, thus holding promise as development areas and migration corridors for target species across diverse future climate scenarios. Consequently, this prioritization strategy not only accomplishes conservation goals cost-effectively but also bolsters the adaptability of the regional rubber ecosystem to forthcoming human-induced disturbances through proactive, forward-thinking planning. It establishes a flexible scientific framework for subsequent dynamic adjustments to protected area boundaries and the amalgamation of carbon sequestration and biodiversity advantages.

## Discussion

### Model analysis

This study’s comparison of five SDM models reveals that different algorithms have their own strengths and weaknesses, and model performance varies depending on the data and context.^29^^,^^30^ GLM models are easy to interpret but struggle to capture complex nonlinear relationships; RF and BRT models offer strong fitting capabilities but are prone to overfitting, resulting in reduced predictive reliability in environments.^31^ In contrast, MaxEnt/MaxNet effectively control model complexity through regularization, achieving a balance between sensitivity and specificity, and are robust when extrapolated to unknown climate conditions.^15^ Therefore, we selected MaxEnt as the primary model for predicting rubber-suitable areas under future climate scenarios, a conclusion supported by other studies.^32^ Although some ensemble models have slightly higher accuracy when extrapolation is not required,^33^^,^^34^ our results indicate that fine-tuned MaxEnt offers comparable accuracy and better generalization, making it suitable for distribution prediction under climate change.^35^

On the other hand, integrating SDM predictions with spatial planning has significant value. Inputting the suitability maps generated by MaxEnt into Marxan optimization can cover most highly suitable habitats at a relatively low cost. Previous studies have demonstrated that using SDM output to guide Marxan effectively covers highly suitable areas and reduces the cost of prioritizing regions.^36^ Our Marxan analysis also indicates that focusing on only approximately 1.9% of the national territory is sufficient to cover the vast majority of potentially suitable areas at a very low cost. This suggests that the combined MaxEnt and Marxan framework can provide scientific and efficient decision-making support for the spatial layout of rubber plantations under climate change.^15^

### Environmental factor analysis

MaxEnt analysis confirmed that isothermality (bio3) and precipitation in the driest month (bio14) are key limiting factors for the suitable distribution of rubber. Rubber growth depends on stable temperatures and adequate water supply during the dry season. Models show that suitability decreases significantly when precipitation in the driest month falls below approximately 60 mm, while greater temperature stability significantly increases suitability. This is consistent with known growth conditions for rubber trees: optimal rubber growth occurs when annual precipitation is approximately 2000–2500 mm and the average annual temperature is 26°C–28°C.^20^ For example, some areas in Guangxi with suitable annual temperatures but low winter and spring precipitation (precipitation in the driest month <80 mm) are rated as having low suitability and require irrigation to compensate for dry season water shortages.^22^ Overall, macroclimate determines the extent of rubber’s suitable habitat, but local factors such as topography and land use influence the specific pattern of suitable habitats. In western Yunnan, areas with predicted suitable climate above 800 m above sea level (such as the Nujiang Gorge) face severe plantation restrictions.^37^ In the Yunnan-Guizhou karst mountainous areas (slopes >25°), even in areas with suitable climate, steep slopes and thin soils make rubber cultivation and soil and water conservation difficult, leading models to overestimate suitability in these areas.^38^ Therefore, climate models should be integrated with topographic and land suitability analyses to improve the feasibility of planning.^39^ Furthermore, because MaxEnt assumes the independence of environmental variables and does not consider the synergistic effects of factors (e.g., slope exacerbates drought), future approaches such as structural equation modeling could be used to more comprehensively analyze the interactions of multiple factors and improve the accuracy of suitable area assessments.

### Future trends and strategies

Comparisons of different climate scenarios indicate that the expansion of the rubber suitable area is most pronounced under the moderate emissions pathway (SSP2-4.5), with more favorable climatic conditions. Moderate warming and increased precipitation meet the growth needs of rubber while avoiding the stress caused by extreme heat and precipitation, resulting in a significant expansion of the highly suitable area. Under the high-emissions scenario (SSP5-8.5), while the average climate tends to be warmer and wetter, expanding the suitable range to some extent, frequent extreme high temperatures and heavy rainfall will impair rubber photosynthesis and yield, slowing the expansion of the suitable area and even reducing it in some areas.^20^^,^^26^ Therefore, from a climate perspective, a moderate-intensity emissions reduction and adaptation scenario is more favorable for the future development of the rubber plantation industry.

However, translating climate potential into actual production capacity requires consideration of non-climatic factors and sustainable management.^40^ Some highly suitable areas predicted by models may not be fully developed due to constraints such as fragmented terrain and water scarcity. When applying future climate trends to guiding planting planning, it is important to assess land carrying capacity, soil conditions, and ecological constraints to avoid land degradation and ecological risks caused by blind expansion. Differentiated policies should be implemented following China’s “14th Five-Year Plan for Natural Rubber Industry Development”: In core production areas such as Hainan and Xishuangbanna (designated “Advantage Zones” in the Plan), industrial layout should be optimized, smart tapping technologies and climate-adaptive varieties promoted, and the Plan’s target of “10% yield increase per unit area” achieved; in expansion-potential areas such as Guangxi and Guangdong (designated “expansion zones”), soil improvement and irrigation infrastructure should be strengthened, rubber-agroforestry pilot projects implemented, and the Plan’s “under-canopy economy” model advanced^41^; in marginal trial areas such as Guizhou and Fujian (designated “reserve zones”), cold-resistant variety trials should be strictly limited to flat land with soil and water conservation measures, following the plan’s principle of “ecology first, prudent advancement.” In all regions, areas with high biodiversity sensitivity should be avoided, buffer areas established between plantations and protected areas, and deforestation for rubber expansion strictly prohibited to ensure simultaneous development and conservation.^42^ Furthermore, socioeconomic factors and extreme climate impacts should be incorporated into future research frameworks, and a comprehensive “climate-ecology-economy” decision-making system established to enhance the resilience and sustainable development of the rubber industry, supporting the Plan’s strategic goal of “increasing natural rubber self-sufficiency by 3 percentage points by 2025.”

### Limitations of the study

Several caveats should be considered when interpreting our findings. First, we relied on a single climate model (CC-CSM2-MR) for future projections; although selected for its skill in East Asia, multi-model ensembles would better characterize scenario uncertainty. Second, the 161 presence records, while spatially thinned to reduce bias, remain modest for national-scale extrapolation; additional field surveys in under-sampled regions (e.g., Guangdong, Fujian) would improve robustness. Third, MaxEnt’s assumption of variable independence may overlook synergistic stressors (e.g., slope exacerbating drought effects), and we did not incorporate extreme climate indices (heatwave frequency, consecutive dry days) directly as predictors. Fourth, Marxan cost layers used planning unit area as a proxy; integrating land opportunity costs or socioeconomic data would enhance practical relevance. Finally, our suitability thresholds (0.09/0.31/0.63) follow common practice but lack crop-specific physiological validation, warranting future ground-truthing.

## Resource availability

### Lead contact

Further information and requests for resources should be directed to and will be fulfilled by the lead contact, Shichuan Yu (linyexiaozu@gmail.com).

### Materials availability

This study did not generate new unique reagents or materials.

### Data and code availability

• Rubber tree occurrence data are available from the Data Center of the Institute of Geographic Sciences and Natural Resources, Chinese Academy of Sciences, the China National Herbarium Resource Center, and the Global Biodiversity Information Facility (GBIF). Field survey data are available from the lead contact upon reasonable request. Environmental datasets: WorldClim 2.1 (https://www.worldclim.org), HWSD (https://www.fao.org/soils-portal), and National Earth System Science Data Center (https://www.geodata.cn).
• All species distribution modeling was performed in R v4.3.2 using the open-source package enmSdmX v1.0 (https://github.com/adamlilith/enmSdmX). Marxan v4.0.6 was obtained from Marxan Solutions (https://marxansolutions.org/software).
• Original code for model comparison, projection, and Marxan input preparation is available at Zenodo/GitHub repository link.
• Any additional information required to reanalyze the data reported in this paper is available from the lead contact upon request.

## Acknowledgments

This study was funded by the Research Start-up Fund (A2032023917) and the Basic Research Project for Young Talents of 10.13039/501100007846Yunnan Provincial Department of Education (KBA3012023045026), the Huang Huasun Expert Workstation of Yunnan Province (grant no. 202305AF150125), the Science and Technology Commissioner Team of Gongxin Township, Mengma Town, Menglian County, Yunnan Province (grant no. 202304BI090032-58), and the National Natural Rubber Industry Technology System - Pu’er Comprehensive Experimental Station (grant no. CARS-33-YN4).

## Author contributions

G.Y., writing – original draft, writing – review and editing, conceptualization, data curation, formal analysis, investigation, methodology, resources, software, validation, and visualization; B.Z., conceptualization, methodology, investigation, resources, and visualization; Y.Z., investigation, data curation, validation, and visualization; L.S., methodology, investigation, data curation, and software; J.Z., investigation, resources, and software; L.T., formal analysis, data curation, and investigation; W.Y., software and investigation; S.Y., writing – review and editing, funding acquisition, data curation, conceptualization, supervision, and project administration; H.D., resources, conceptualization, methodology, investigation, software, funding acquisition, and psssroject administration.

## Declaration of interests

The authors declare no conflicts of interest.

## Declaration of generative AI and AI-assisted technologies in the writing process

During the preparation of this work, the authors did not use any generative AI or AI-assisted technologies in the writing process.

## STAR★Methods

### Key resources table

**Table:** 

REAGENT or RESOURCE	SOURCE	IDENTIFIER
**Deposited data**		

bioclimatic data	WorldClim 2.1	https://www.worldclim.org
HWSD soil data	FAO/IIASA/ISRIC/ISSCAS/JRC	https://www.fao.org/soils-portal
GBIF occurrence data	GBIF Secretariat	https://www.gbif.org
National land use data	National Earth System Science Data Center	https://www.geodata.cn/
National population distribution data	National Earth System Science Data Center	https://www.geodata.cn/

**Software and algorithms**		

R v4.3.2	R Core Team	https://www.r-project.org/
Marxan v4.0.6	Marxan Solutions	https://marxansolutions.org/software
ArcGIS Pro v3.1	Environmental Systems Research Institute	https://www.esri.com/arcgis-pro
enmSdmX v1.0	GitHub	https://github.com/adamlilith/enmSdmX.

**Other**		

Rubber tree field survey data	Field survey	Available from lead contact

### Experimental model and study participant details

This study does not use experimental models typical in the life sciences. This section is omitted per journal guidelines.

### Method details

#### Study area

This is the main distribution area for rubber tree plantations in China and also a potential suitable expansion area for the future.^41^ The study area is located between 18°N and 27°N latitude and 98°E and 120°E longitude. The terrain is complex, including plains, hills, and some low mountainous areas. The soil types are primarily red soil, brick-red soil, and yellow soil, which are suitable for rubber tree growth. The climate is characterized by tropical monsoon and subtropical monsoon climates, with an annual average temperature of 20°C to 27°C and annual precipitation of 1,000 to 2,000 mm. Future climate change may significantly impact the suitable distribution within the region. The natural vegetation in the study area is primarily tropical rainforests and subtropical evergreen broad-leaved forests. Rubber trees are an important tropical cash crop in China and play a significant role in local economic development. However, their expansion has also led to land use conflicts and ecological and environmental pressures.^43^ Selecting this study area is of great significance, as it can provide data support for the scientific planning and ecological protection of rubber plantations under future climate change scenarios, as well as decision-making basis for regional economic development and ecological sustainability.^44^

#### Species occurrence data

**Figure 12:**
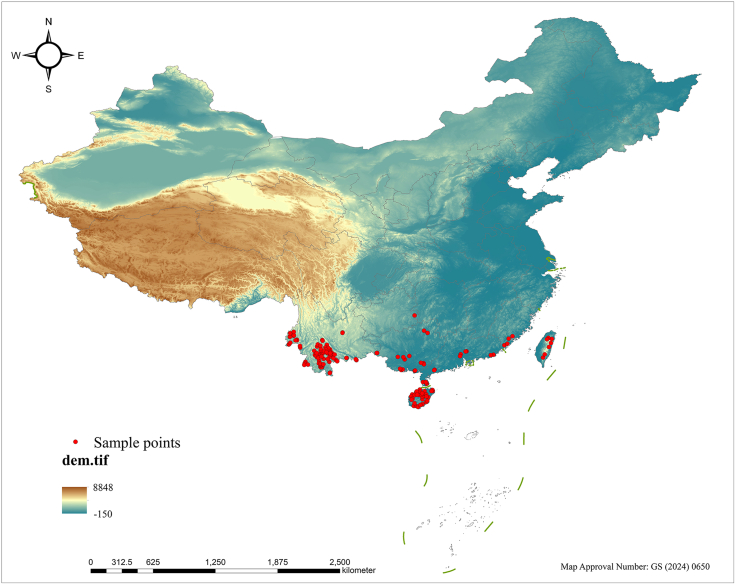
Schematic diagram of sample points for rubber tree distribution Records were compiled from the Data Center of the Institute of Geographic Sciences and Natural Resources (Chinese Academy of Sciences), the China National Herbarium Resource Center (PE), the Global Biodiversity Information Facility (GBIF), and field surveys conducted in Pu’er, Yunnan Province (November 2024). The majority of points cluster in southern Yunnan (Xishuangbanna and Pu’er regions), Hainan Island, southwestern Guangxi, southern Guangdong (Leizhou Peninsula), and southern Taiwan, reflecting the current northern limit of commercial rubber cultivation in China (18°–24°N).

The data were compiled from the Data Center of the Institute of Geographic Sciences and Natural Resources, Chinese Academy of Sciences, the China National Herbarium Resource Center, the Global Biodiversity Information Facility (GBIF), and published literature, supplemented by a field survey of rubber plantations conducted in Pu’er, Yunnan Province in November 2024. The data yielded 179 precise longitude and latitude distribution records of rubber trees (*Hevea brasiliensis*), primarily concentrated in Yunnan, Guangxi, Guangdong, and Hainan.Spatial thinning (minimum 10 km separation via enmSdmX::geoThin) reduced this to 161 records to mitigate sampling bias (Figure 12).^45^

#### Environmental variables

**Figure 13:**
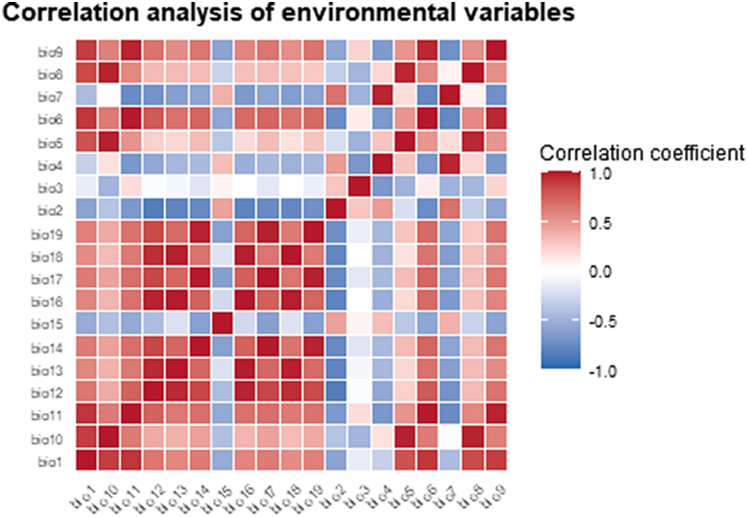
Correlation matrix of 19 bioclimatic variables Dark red indicates high correlation (|r| > 0.8). Variables exceeding this threshold were sequentially removed; eight non-correlated variables (bio1, bio3, bio4, bio6, bio7, bio9, bio11, and bio14) were retained for final modeling, ensuring predictor independence.

Environmental variables include climate, topography, soil composition, socioeconomic conditions, and land-use change. Soil pH, organic matter content, and related data were obtained from the Human World Soil Database (HWSD). National land use data from 1990 to 2015 (resolution 1000×1000) and national population distribution data from 2010 to 2020 (resolution 1000×1000) were obtained from the National Earth System Science Data Center(Hyndman et al., 2025). Digital elevation model (DEM) terrain data with a resolution of 1000×1000 were downloaded from the Geospatial Data Cloud. Historical climate data from 1970 to 2000 were obtained from the World Climate Database, covering 19 key climate variables that influence species distribution. All bioclimatic data are standardized and dimensionless at a resolution of 2.5′ (equivalent to 5000 m × 5000 m). Next, an ArcGIS masking technique was used to extract a data layer of all environmental variables within China. The coordinate system was standardized to the GCS WGS 1984 geographic coordinate system, with a spatial resolution of 1000 m × 1000 m. Neighborhood interpolation was used to fill missing values in the raster data to ensure data integrity and consistency. Correlation analysis was performed on 19 climate variables to eliminate highly correlated environmental variables, with Figure 13 visually confirming the |r| > 0.8 threshold—retained variables (bio1, bio3, bio4, bio6, bio7, bio9, bio11, bio14) show no dark red correlations, while excluded variable pairs exceed this threshold, ultimately retaining eight key factors with ensured predictor independence. Furthermore, to characterize the dynamic impact of human activities on areas potentially suitable for rubber cultivation, we temporally resampled land use/cover data (LUCC) at 1000-meter resolution for six periods from 1990 to 2015 and population spatial distribution data (Pop) at 1000-meter resolution from 2010 to 2020. Land use data (1990–2015) and population data (2010–2020) were selected based on data availability and rubber plantation expansion dynamics. The 1990–2015 period captures China’s rapid rubber expansion phase,^9^ while 2010–2020 population data align with the most recent national census cycles. We calculated transition matrices at 5-year intervals for land use to detect gradual landscape changes, and 10-year intervals for population to capture demographic shifts. Temporal representativeness was validated by comparing change rates across intervals—stable trends (CV < 15%) confirmed representative periods. The 1990–1995 and 2010–2015 intervals were selected as baseline and current human activity intensities, respectively. Finally, all raster data were stored for subsequent scenario simulations using the species distribution model (SDM) and Marxan.

#### Future climate projections

Future climate scenarios are based on three socioeconomic pathways: sustainable development (SSP126), moderate emissions (SSP245), and high emissions (SSP585). The CC-CSM2-MR model is used, which is known for its accurate simulation of climate patterns in China and surrounding areas.^46^

We used the CC-CSM2-MR global climate model under three CMIP6 SSP scenarios (SSP126, SSP245, SSP585) for three periods: 2041–2060, 2061–2080, and 2081–2100.

#### Species distribution modeling

All species distribution models (SDMs) were executed in R version 4.3.2^47^ using the open-source enmSdmX package,^48^ which provides a standardized interface for various algorithms including Random Forest (RF), Generalized Linear Model (GLM), Boosted Regression Trees (BRT), MaxEnt, and MaxNet. To effectively mitigate sampling bias and improve result reliability,^45^ occurrence records were first spatially thinned using enmSdmX:geoThin, and a spatially explicit 4-fold cross-validation was implemented using geoFold to partition presence and absence records (Table 1). RF, GLM, BRT, MaxEnt, and MaxNet were then concurrently trained using the respective enmSdmX functions (trainRF, trainGLM, trainBRT, trainMaxEnt, and trainMaxNet) with identical environmental variables and sample partitions. The complexity of each algorithm was optimized through automatic parameter tuning via the trainByCrossValid function based on AICc and cross-validation error, with manual adjustment of k-fold configurations.

#### Suitability classification

Using ArcGIS, MaxEnt simulations were employed to predict suitable areas for rubber plantations under three socioeconomic pathways and climate scenarios (2041–206, 2061–2080, and 2081–2100). The prediction results were reclassified into four categories based on suitability levels: unsuitable (<0.09), low suitability (0.09–0.31), moderate suitability (0.31–0.63), and high suitability (>0.63), integrating geographical distribution data. The jackknife method was utilized to analyze influencing factors.^49^^,^^50^ Subsequently, a reclassification operation was conducted in ArcGIS, assigning values (unsuitable area = 1, low suitability area = 2, moderate suitability area = 3, high suitability area = 4) based on the established criteria. By integrating raster dataset attributes and conducting statistical analyses, the areas of different suitability areas were determined, reflecting the distribution of rubber plantations across varying climatic conditions.

#### Spatial prioritization with Marxan

The Marxan tool (Marine reserve design using spatially explicit annealing) is actively maintained and freely accessible on the official Marxan Solutions website.^51^ the cost of each 2 km × 2 km planning unit (PU) was defined as its area, and this value was explicitly assigned to the cost field in the pu.dat input file for Marxan. This ensures that the model has a quantified cost basis for conducting cost-benefit optimization. The rubber suitability grid, a continuous probability range of 0–1 generated by MaxEnt under the SSP245 scenario for 2081–2100, was converted into a binary format. The probability values for each planning unit were extracted and transformed into a vector layer, designated as a singular conservation feature in Marxan, denoted as “rubber_suitable.” The contribution value of each PU to this feature corresponds to the probability of occurrence within that PU. The area within the unit represents the conservation cost of the PU. To determine the optimal parameters, we tested SPF values (1, 5, 20, 100) based on target achievement and cost efficiency, finding that SPF=5 achieved the 30% target at the lowest cost (15.65) without excessive boundary inflation. We evaluated BLM values (0.01, 1, 10) through a trade-off between boundary length and compactness, with BLM=1 providing optimal aggregation without over-aggregation. After multiple calibrations, the “BLM1 target300 spf5” solution was selected as the best balance between meeting the target and minimizing cost. The objective was to retain 30% of the total area of future suitable areas, corresponding to various SPF and BLM values, to strike a balance between compactness and cost. The process involved 100 Repeat Runs using simulated annealing with 1,000,000 iterations for iterative improvement. The best solution was then converted into a vector layer to identify the optimal spatial distribution for priority development under the most favorable scenario.

### Quantification and statistical analysis

#### Model evaluation

Performance was assessed using Area Under the Receiver Operating Characteristic Curve (AUC), True Skill Statistic (TSS), and Cohen’s Kappa, computed on independent test folds from spatial cross-validation. AUC measures discrimination capacity (1 = perfect); TSS = sensitivity + specificity − 1, ranging from −1 to 1; Kappa accounts for chance agreement. The training-test gap (ΔAUC) was used to diagnose overfitting.^52^

#### Variable importance

Permutation importance was extracted from MaxEnt jackknife tests. Response curves were evaluated for mechanistic plausibility against published physiological thresholds.

#### Area statistics

Suitable areas were calculated in ArcGIS Pro by multiplying pixel counts by pixel area (1 km^2^), aggregated by suitability class and scenario. Percentage changes were calculated relative to current conditions.

#### Statistical software

R v4.3.2 (R Core Team, 2023); packages: enmSdmX v1.0, dismo, raster, sf. Marxan v4.0.6. ArcGIS Pro 3.1 for spatial processing.

### Additional resources

STAR Methods guide: https://marlin-prod.literatumonline.com/pb-assets/journals/research/cell/methods/Methods_Guide_general.
